# An interphase pool of KIF11 localizes at the basal bodies of primary cilia and a reduction in KIF11 expression alters cilia dynamics

**DOI:** 10.1038/s41598-020-70787-4

**Published:** 2020-08-18

**Authors:** Abigail A. Zalenski, Shubhra Majumder, Kuntal De, Monica Venere

**Affiliations:** 1 Department of Radiation Oncology, James Cancer Hospital and Comprehensive Cancer Center, The Ohio State University Wexner School of Medicine, 440 Tzagournis Medical Research Facility, 420 West 12th Avenue, Columbus, OH 43210 USA; 2 Neuroscience Graduate Program, The Ohio State University, Columbus, OH 43210 USA; 3 Present Address: Department of Life Sciences and the School of Biotechnology, Presidency University, Kolkata, 700073 India; 4 Present Address: Bioscience Division, Oak Ridge National Lab, Oak Ridge, TN 37830 USA

**Keywords:** Cancer, Cell biology

## Abstract

KIF11 is a homotetrameric kinesin that peaks in protein expression during mitosis. It is a known mitotic regulator, and it is well-described that KIF11 is necessary for the formation and maintenance of the bipolar spindle. However, there has been a growing appreciation for non-mitotic roles for KIF11. KIF11 has been shown to function in such processes as axon growth and microtubule polymerization. We previously demonstrated that there is an interphase pool of KIF11 present in glioblastoma cancer stem cells that drives tumor cell invasion. Here, we identified a previously unknown association between KIF11 and primary cilia. We confirmed that KIF11 localized to the basal bodies of primary cilia in multiple cell types, including neoplastic and non-neoplastic cells. Further, we determined that KIF11 has a role in regulating cilia dynamics. Upon the reduction of KIF11 expression, the number of ciliated cells in asynchronously growing populations was significantly increased. We rescued this effect by the addition of exogenous KIF11. Lastly, we found that depleting KIF11 resulted in an increase in cilium length and an attenuation in the kinetics of cilia disassembly. These findings establish a previously unknown link between KIF11 and the dynamics of primary cilia and further support non-mitotic functions for this kinesin.

## Introduction

KIF11, also known as Eg5 and Kinesin-5, is a mitotic kinesin that is responsible for forming and maintaining the bipolar spindle. KIF11 has primarily been studied for its mitotic role, and is thought to be mostly degraded after mitosis^1–6^. However, there is a growing body of work that implicates KIF11 in other, non-mitotic cell processes. KIF11 has been demonstrated to have microtubule polymerase activity, as well as to control axon growth^7,8^. It has also been shown to mediate centrosome migration after mitosis and move Golgi material^3,9^. Additionally, we have previously studied a non-mitotic role for KIF11 in the context of glioblastoma (GBM). We identified that in the GBM cancer stem cell subpopulation, KIF11 is highly overexpressed throughout the cell cycle due to attenuated protein degradation^4,10^. Cancer stem cells are found in many tumor types, and are characterized as being radio and chemo resistant. We found that this interphase pool of KIF11 was responsible for driving tumor cell invasion and process formation^4^.

In addition to these reported non-mitotic roles of KIF11, there are two patient populations that have reported mutations in the *KIF11* gene. The associated diseases are known as microcephaly with or without chorioretinopathy, mental retardation, and lymphedema (MCLMR) and familial exudative vitreoretinopathy (FEVR), with the latter having reported *KIF11* mutations in only a subset of the patients. The mutations are found throughout the gene, and a given mutation is not typically seen in more than one patient unless inherited^11–16^. Importantly, all reported patient mutations are heterozygous, with many of the mutations predicted to lead to haploinsufficiency of KIF11^11,13,16^. These patients share a characteristic collection of phenotypes, but not all patients present with every phenotype, and some are more severe than others. These phenotypes include those mentioned in the disease names, such as microcephaly and mental retardation, but also include characteristics such as retinopathy, lissencephaly, syndactyly, and cerebellar hypoplasia^11,16^. Interestingly, these symptoms share a significant overlap with the collection of symptoms found in patients with ciliopathies^17,18^. Ciliopathies occur as a result of mutations in critical primary cilia genes. The primary cilium is a sensory organelle that can project from many different cell types, and is required for developmental signaling pathways such as sonic hedgehog^17,18^. Despite these overlapping symptoms, KIF11 has never been implicated as a regulator of primary cilia. However, a recent publication that studied MCLMR and FEVR patients demonstrated that KIF11 is found in photoreceptor cilia, and is implicated as a driver of retinal ciliopathy^19^. Photoreceptor cilia are a modified form of primary cilia, so despite this report, the question still remains if KIF11 is found at other primary cilia and if it has a role in the regulation of this organelle^20^. The presence of KIF11 outside of mitosis, combined with the patient mutations and phenotypes, led us to hypothesize that KIF11 plays a role in regulating primary cilia dynamics.

In this study, we sought to determine if KIF11 is present at primary cilia in several different cell types, as well as to begin to understand the function of KIF11 at primary cilia. Using two different approaches, we showed that reducing KIF11 levels impacted both cilia dynamics and cilia length. Our findings represent the first step in understanding this previously undescribed role for KIF11.

## Results

### KIF11 localizes to the basal body of primary cilia in GBM cells

**Figure 1:**
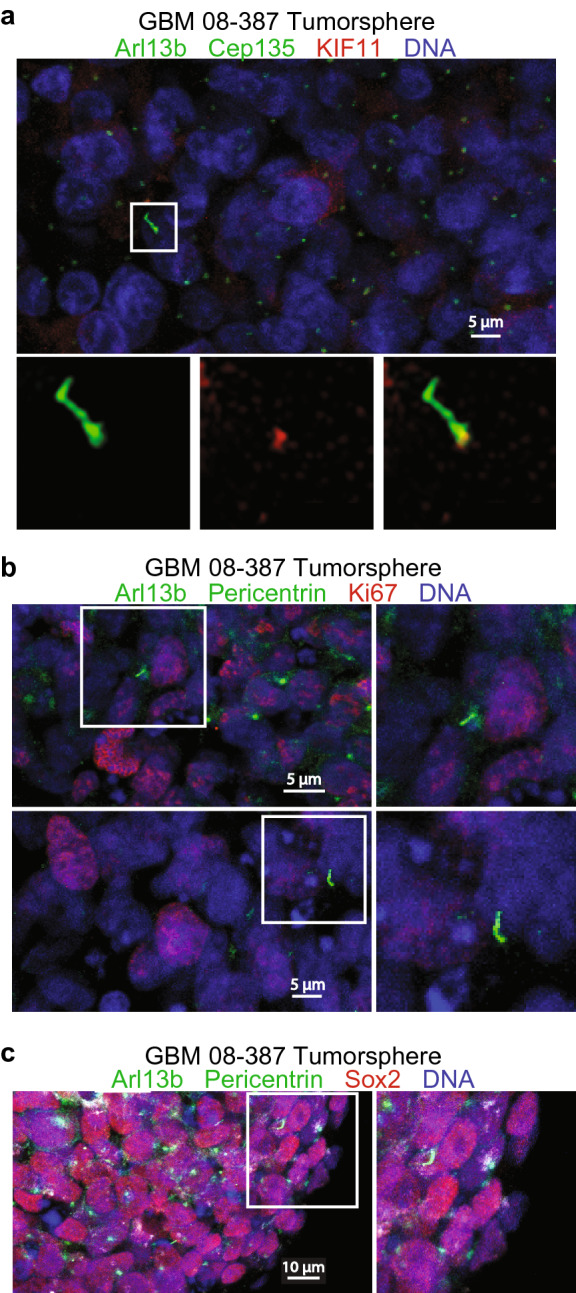
KIF11 localizes to the basal body of primary cilia in patient-derived glioblastoma tumorspheres. (**a**) Tumorspheres from GBM 08-387 were fixed and probed with α-Arl13b (green), α-Cep135 (green), α-KIF11 (red), and the DNA was counter-stained with Hoechst (blue). (**b**) 08-387 tumorspheres were fixed and probed with α-Arl13b (green), α-Pericentrin (green), α-Ki67 (red), and the DNA was counter-stained with Hoechst (blue). (**c**) 08-387 tumorspheres were fixed and probed with α-Arl13b (green), α-Pericentrin (green), α-Sox2 (red), and the DNA was counter-stained with Hoechst (blue). White boxes in a-c represent regions of interest shown in the additional panels.

Our previous work with KIF11 demonstrated that the protein is overexpressed throughout the cell cycle in patient-derived GBM cells^4^. Additionally, it has been demonstrated that primary cilia can be found in patient-derived GBM samples, but the role of these cilia is somewhat disputed^21,22^. Because of the elevated levels of KIF11, and the presence, albeit not well understood, of primary cilia in GBM tumorspheres, we sought to understand if KIF11 was associated with these primary cilia. To answer this question, we grew patient-derived GBM (08-387) tumorspheres in suspension, then fixed the spheres and immunolabeled for KIF11 and the primary cilia markers Arl13b and Cep135 to mark both the axoneme and basal body/centrosome, respectively. Confocal imaging of cilia in tumorspheres revealed that KIF11 localized near the basal bodies of primary cilia (Fig. 1a). Because the role of primary cilia in GBM tumorspheres is not well understood, we also aimed to further characterize the ciliated cells in these samples. We first immunolabeled cells for Ki67 (a marker for proliferating cells), Arl13b, and pericentrin (a marker for pericentriolar material). Imaging of these tumorspheres demonstrated that primary cilia could be found on both Ki67 negative and positive cells (Fig. 1b). This finding is interesting, as primary cilia are most often found on quiescent cells, and hence would not stain positively for Ki67^23^. However, there are examples where cilia are present on proliferative cell populations, such as on neural progenitors in fetal and postnatal proliferative zones, as well as in cancer cells^21,22,24,25^. Our data agrees with previous reports that cilia can be present on proliferating cancer cells. We also labeled tumorspheres for Sox2 (a marker for cancer stem cells), Arl13b, and pericentrin to determine if cilia are present on cancer stem cells. We found that almost all ciliated cells were Sox2 positive, indicating a possible association between primary cilia and the cancer stem cell population (Fig. 1c). These data demonstrate that KIF11 is localized at the basal bodies of primary cilia in GBM tumorspheres. Furthermore, they support previous reports that primary cilia in GBM tumorspheres have been identified on Ki67 positive cells^21^.

### KIF11 localizes to the basal body of primary cilia in non-neoplastic cells and associates with ciliary proteins

**Figure 2:**
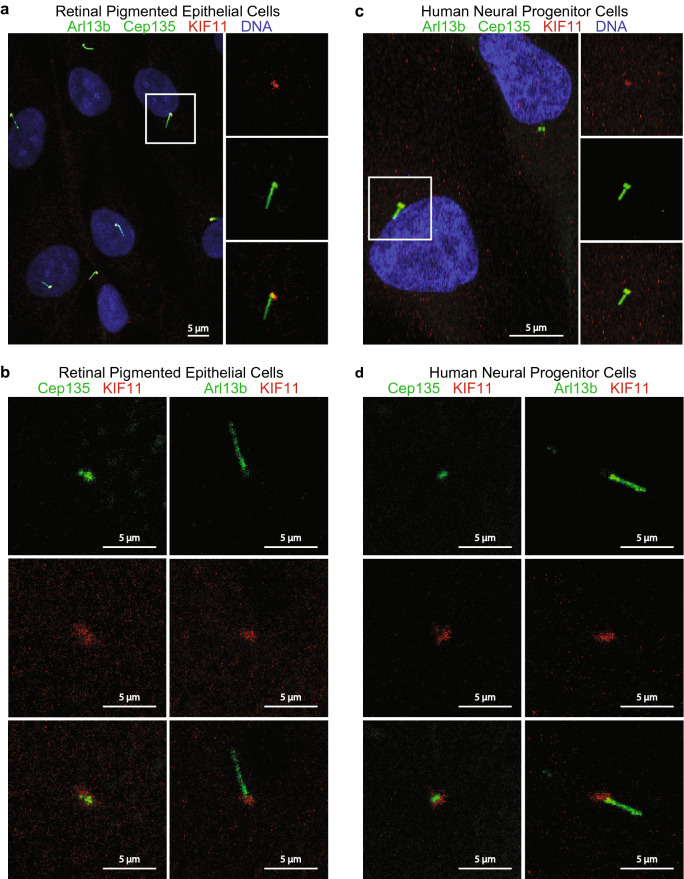
KIF11 is localized to the basal body of primary cilia in retinal pigmented epithelial cells and immortalized human neural progenitor cells. (**a**) Serum starved RPE1 cells were fixed and probed with α-Arl13b (green), α-Cep135 (green), α-KIF11 (red), and the DNA was counter-stained with Hoechst (blue). (**b**) Serum starved RPE1 cells were fixed and probed with α-Cep135 (green) and α-KIF11 (red) (left images) and α-Arl13b (green) and α-KIF11 (red) (right) to highlight KIF11 localization at the basal body. (**c**) Human neural progenitor cells were fixed and probed with α-Arl13b (green), α-Cep135 (green), α-KIF11 (red), and the DNA was counter-stained with Hoechst (blue). (**d**) Human neural progenitor cells were fixed and probed with α-Cep135 (green) and α-KIF11 (red) (left images) and α-Arl13b (green) and α-KIF11 (red) (right) to highlight KIF11 localization at the basal body. White boxes in (**a**) and (**c**) represent regions of interest shown in the additional panels.

As primary cilia in tumorspheres are not well understood and may be differently regulated than primary cilia in non-neoplastic cells, we next wanted to investigate whether KIF11 also localized to basal bodies of primary cilia on non-neoplastic cells. To address this question, we collected a serum starved population of immortalized retinal pigmented epithelial cells (hTERT-RPE1 or RPE1 cells), and immunolabeled for KIF11, Arl13b, and Cep135. We immunolabeled these antibodies simultaneously and each antibody individually to validate KIF11 localization distinctly at the basal body region of primary cilia. Serum starvation in RPE1 led to quiescent/ G0-synchronized cells, which were used to maximize the number of cells with primary cilia in our samples. Akin to what we identified in GBM cancer stem cells, we found that KIF11 was also localized near the basal body of primary cilia in RPE1 cells (Fig. 2a,b). We also used another cell model, immortalized human neural progenitor cells, to examine KIF11 and primary cilia. Again, using immunofluorescence, we determined that KIF11 localized near the basal body of these primary cilia as well (Fig. 2c,d). Lastly, we collected G0-synchronized RPE1 cells for proteomic analysis to determine if KIF11 interacted with any cilia-associated proteins. We immunoprecipitated KIF11 from our samples, and then performed proteomic analyses to identify any interacting partners. We identified over 30 cilia-associated proteins within the interactome for KIF11 in G0-synchronized RPE1 cells; 14 of these proteins were identified in replicate screens (Table 1, bold indicates proteins identified in replicate screens, non-bolded identified in one screen). These results demonstrate that KIF11 is localized to primary cilia in multiple cell types and may directly or indirectly interact with cilia-associated proteins, suggesting that KIF11 may play a role in the regulation of cilia dynamics in normal cells.

**Table 1:** KIF11 associated primary cilia proteins.

KIF11 interactor	Cilia-related protein function	References
**KIF5B/Kinesin-1**	Associated with regulating cilia length through interaction with BBSome	^26,27^
**HSPA8**	Chaperone, associated with photoreceptors	^20,27^
**SPTAN1**	Cilia associated, knockdown decreases number of cilia	^27,28^
**FLNA**	Ciliogenesis and basal body positioning; associates with Meckelin (gene causing MKS)	^29,30^
**IPO5**	Enriched in cilia	^27,30^
**MYH10**	Responsible for centriole migration during formation of primary cilia	^27,31,32^
**SF3B2**	Identified in primary cilia interaction screen	^27^
**PKM**	Identified in primary cilia interaction screen	^27^
**SF3A1**	Identified in primary cilia interaction screen	^27^
**HSP90AB1**	Identified in primary cilia interaction screen	^27^
**EFTUD2**	Identified in primary cilia interaction screen	^27^
**EEF1A1**	Identified in primary cilia interaction screen	^27^
**ATP2A2**	Identified in primary cilia interaction screen	^27^
**CLTC**	Identified in primary cilia interaction screen	^27^
SEPT2	Associated with cilia length modulation	^27,33,34^
SEPT9	Associated with cilia length modulation	^27,33,34^
DCTN1	Associated with ciliogenesis	^27,35^
EXOC4	Centrosome component	^27,36^
ANXA1	Enriched in cilia	^27,30^
ATP2B4	Localizes to/associated with primary cilia	^27^
GSN	Promotes cilia formation	^27^
CTNNB1	Wnt pathway effector	^37,38^
HSP90B1	Identified in primary cilia interaction screen	^27^
HK1	Identified in primary cilia interaction screen	^27^
TUBA1B	Identified in primary cilia interaction screen	^27^
HSP90AA1	Identified in primary cilia interaction screen	^27^
TUBB4B	Identified in primary cilia interaction screen	^27^
DNM2	Identified in primary cilia interaction screen	^27^
CASK	Identified in primary cilia interaction screen	^27^
TUBB	Identified in primary cilia interaction screen	^27^
TUBA1A	Identified in primary cilia interaction screen	^27^

### KIF11 depletion alters cilia dynamics and length

**Figure 3:**
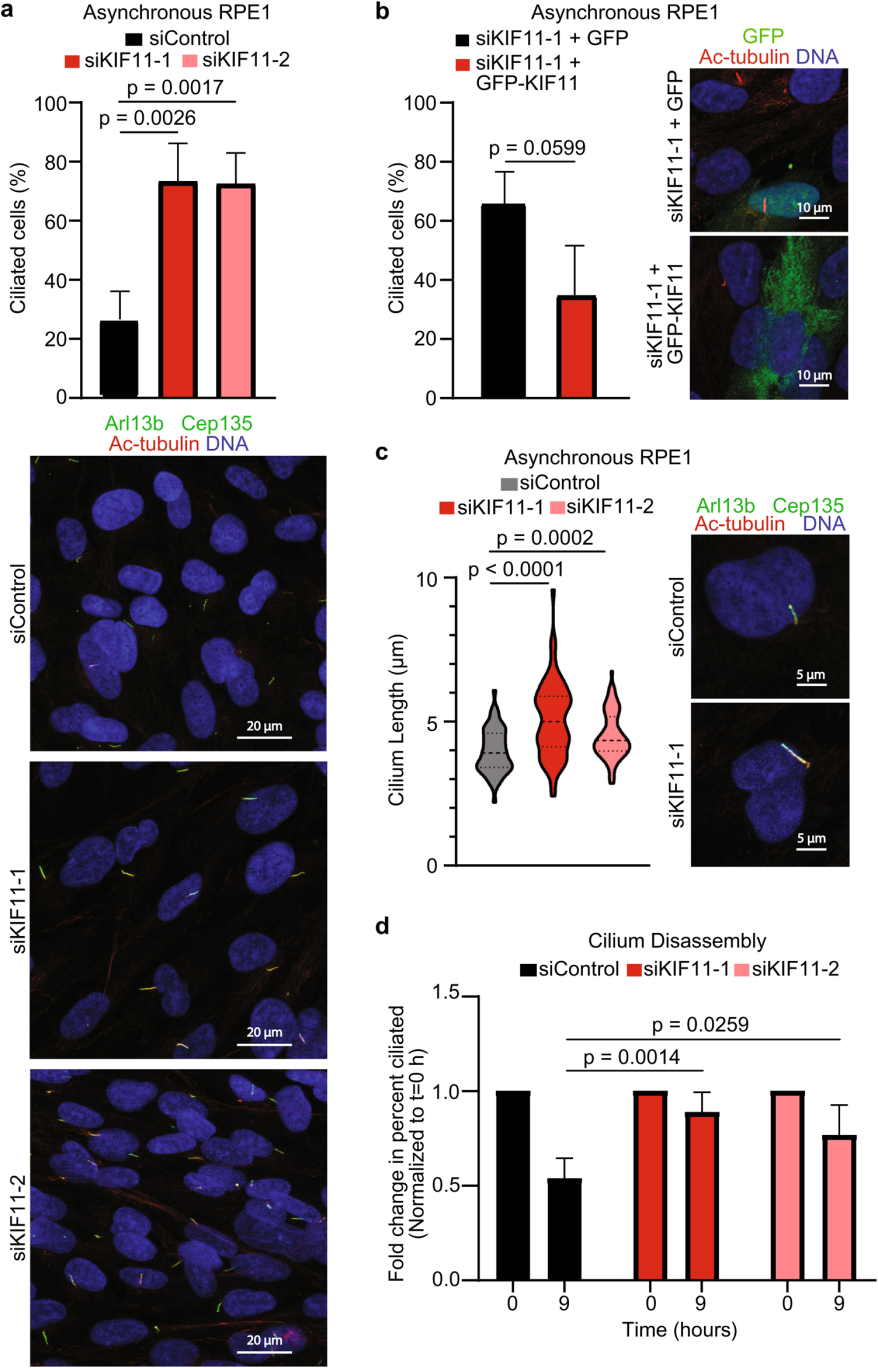
Depleting KIF11 leads to alterations in cilia dynamics and length. (**a**) Asynchronous RPE1 cells were treated with either siControl, siKIF11-1, or siKIF11-2. After 72 h, cells were fixed and probed with α-Arl13b (green), α-Cep135 (green), α-KIF11 (red), and the DNA was counter-stained with Hoechst (blue). Percentage of ciliated cells was calculated for each condition and graphed (n = 3 biological replicates with 150–250 cells counted per n). Data analyzed by Student’s *t*-test. Error bars represent standard deviation. Representative images for each condition are shown in the image panels. (**b**) Asynchronous RPE1 cells were treated with siKIF11-1, and after 24 h were then subsequently transfected with GFP, or GFP-KIF11. 48 h later cells were fixed and probed with α-Arl13b (red), α-Cep135 (red), α-GFP (green), and the DNA was counter-stained with Hoechst (blue). Percentage of ciliated, GFP-positive cells was calculated from the total number of GFP positive cells for each condition, and results were graphed (n = 3 biological replicates with 50–100 cells counted per n). Data analyzed by Student’s *t*-test. Error bars represent standard deviation. Representative images for each condition are shown in the image panels. (**c**) Cilium length was measured for each condition and graphed (n = 3 biological replicates with 20–30 cells counted per n). Data analyzed using a Student’s *t*-test. Error bars represent standard deviation. Median and quartiles denoted by dashed lines within violin plots. Representative images for each condition are shown in the image panels. (**d**) To evaluate disassembly of primary cilia, asynchronous RPE1 cells were treated with either siControl, siKIF11-1, or siKIF11-2. After 24 h, cells were serum starved. 24 h later cells from each condition were collected, and serum was added back to the remaining cells. Cells were collected 9 h later. All cells were fixed and probed with α-Arl13b, α-Cep135, α-KIF11, and the DNA was counter-stained with Hoechst. Percentage of ciliated cells was calculated for each condition and graphed (n = 3 biological replicates with 125–250 cells counted per n). Data analyzed by 2-way ANOVA with Sidak’s post-test. Error bars represent standard deviation.

Having identified that KIF11 localized at the basal body of primary cilia and interacts with ciliary proteins, we next sought to investigate if KIF11 had a role in regulating primary cilia. To answer this question, we used two siRNAs designed against KIF11 (siKIF11-1 and siKIF11-2), as well as a control siRNA (siControl) with depletion of KIF11 confirmed at the protein level by immunoblotting (Supplementary Fig. 1a,b). We treated asynchronously growing RPE1 cells with either siControl, or one of the two siKIF11s. siRNAs to KIF11 were titrated to an amount that did not impact the ability of the cells to go through the cell cycle, as demonstrated by equal numbers of EdU positive cells in siControl and siKIF11 groups (Supplementary Fig. 1c). 72 h post siRNA treatment, cells were fixed and immunofluorescence was performed to examine primary cilia. KIF11 depletion led to a significant increase in the number of ciliated RPE1 cells over siControl-treated cells (Fig. 3a). Importantly, this shift towards increased ciliation was not merely due to all of the cells becoming quiescent, as evidenced by the incorporation of EdU into an equal number of cells as those treated with siControl (Supplementary Fig. 1c). Since a decrease in KIF11 via siRNA caused an increase in the number of primary cilia, we also overexpressed GFP and GFP-KIF11 in asynchronous RPE1 cells to determine if this would also impact the number of primary cilia. Both 24 and 48 h after GFP or GFP-KIF11 transfection into RPE1 cells, we did not see any difference in the percent of ciliated cells between the two groups (Supplementary Fig. 1d). We then co-transfected siKIF11-1 with either GFP alone, or an siRNA-resistant, GFP-tagged KIF11 to determine if KIF11 was sufficient to rescue this change in the percentage of ciliated cells. When GFP-KIF11 was transfected into the cells, the phenotype of increased cilia was partially rescued in positively-transfected cells (Fig. 3b). We also determined that depleting KIF11 increased the length of primary cilia over siControl-treated cells (Fig. 3c). The length of primary cilia is tightly regulated, and changes in length could indicate changes in cilia-mediated signaling^39,40^. To further explore how reducing KIF11 impacted primary cilia dynamics, we studied the impact on serum-induced primary cilia disassembly. When RPE1 cells are serum-starved, approximately 80% of the cells will become quiescent and form cilia. In this experiment, RPE1 cells were serum-starved for 48 h, and then serum was added back to the cells to induce cilium disassembly and cell cycle reentry. Cells were fixed for immunofluorescence at the time of serum addition (0 h), as well as 9 h later, when cilia in more than 50% of the cells should have been disassembling. At 9 h post-serum addition, there were significantly more ciliated cells remaining when cells were treated with siKIF11 (Fig. 3d). Together, these data demonstrate, for the first time, that a reduction in KIF11 is enough to alter primary cilia dynamics and presence on a cell, as well as primary cilia length. Furthermore, we have shown that this reduction in KIF11 is permissible in the context of mitosis, but there is a separate, primary cilium-specific effect that cannot function normally with the decrease in protein.

### Heterozygous *KIF11* alters cilia dynamics and length

**Figure 4:**
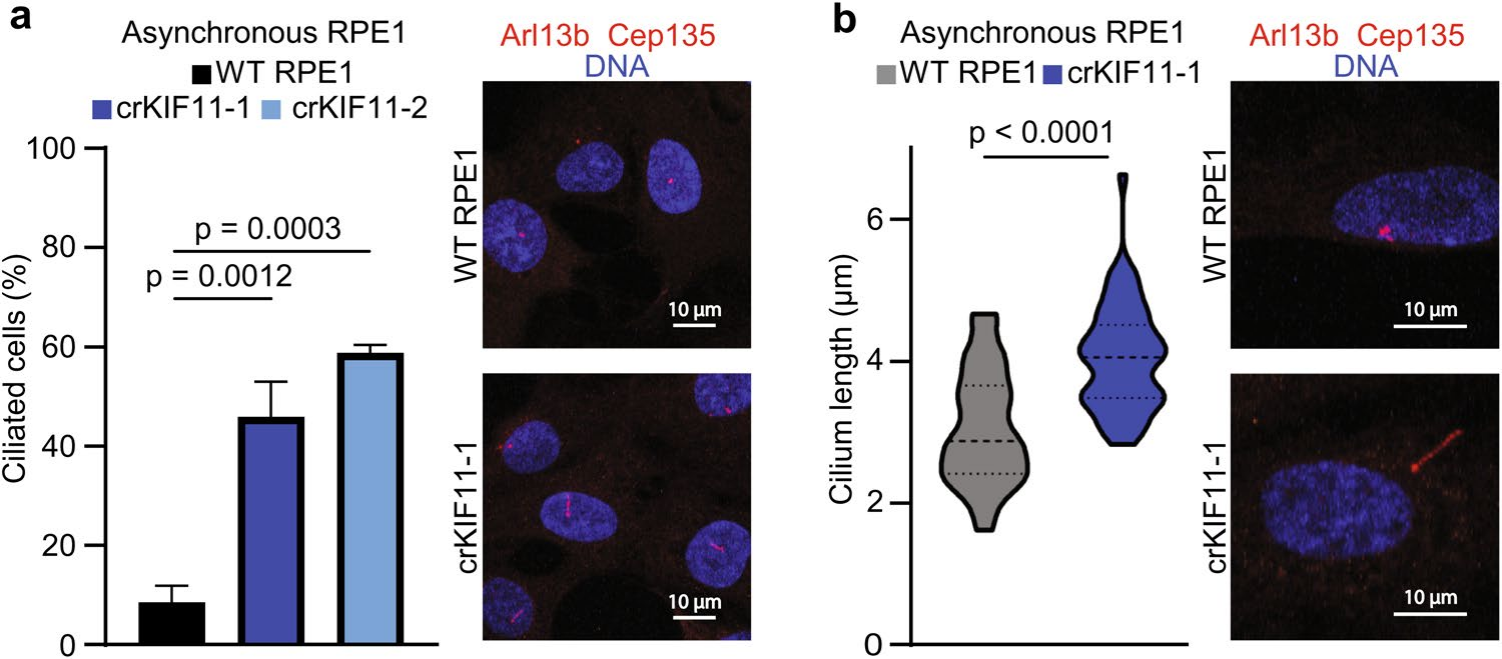
Heterozygous *KIF11* leads to alterations in cilia dynamics and length. (**a**) Asynchronously growing wild type (WT) RPE1 and heterozygous crKIF11 cells were fixed and probed with α-Arl13b (red), α-Cep135 (red), and the DNA was counter-stained with Hoechst (blue). Percentage of ciliated cells was calculated for each condition and graphed (n = 3 biological replicates for WT RPE1 and crKIF11-1 with 90–100 cells counted per n, n = 2 biological replicates for crKIF11-2 with 90–100 cells counted per n). Data analyzed by Student’s *t*-test. Error bars represent standard deviation. Median and quartiles denoted by dashed lines in violin plots. Representative images for each condition are shown in the image panels. (**b**) Cilium length was measured for each condition and graphed (n = 3 biological replicates with 20–30 cells counted per n). Data analyzed using a Student’s *t*-test. Error bars represent standard deviation. Representative images for each condition are shown in the image panels.

Because MCLMR and FEVR patients are reported to have a mutation in only one allele of *KIF11*, we next created two heterozygous CRISPR/Cas9 clonal lines of RPE1 cells to experimentally represent this genetic state (crKIF11-1 and crKIF11-2). Gene editing via CRISPR/Cas9 more closely reflects the patient mutational state than RNA interference via siRNA, and hence is critical for evaluating the impact of reduced KIF11 on primary cilia^12–14,16,41^. Depletion of KIF11 was confirmed by immunoblotting (Supplementary Fig. 2a–c). Asynchronously growing wild type RPE1 cells, as well as asynchronous crKIF11-1 and crKIF11-2 cells were collected for immunofluorescence. Similar to our siRNA data, crKIF11-1 and crKIF11-2 cells were significantly more ciliated than wild type RPE1 cells (Fig. 4a). We also confirmed by EdU labeling that crKIF11 cells were still cycling and had similar numbers of EdU positive cells as wild type RPE1 (Supplemental Fig. 2d). Furthermore, we monitored cell growth via the Incucyte ZOOM live-cell imaging system, and again found similar growth rates between wild type RPE1 and crKIF11 cells (Supplemental Fig. 2e). Lastly, we found that cilium length was significantly increased in crKIF11 cells over wild type RPE1 (Fig. 4b). Again, these results suggest that reducing KIF11 can alter cilia dynamics, without disrupting its ability to go through mitosis, indicating that there is a primary cilia-specific role for KIF11.

## Discussion

**Figure 5:**
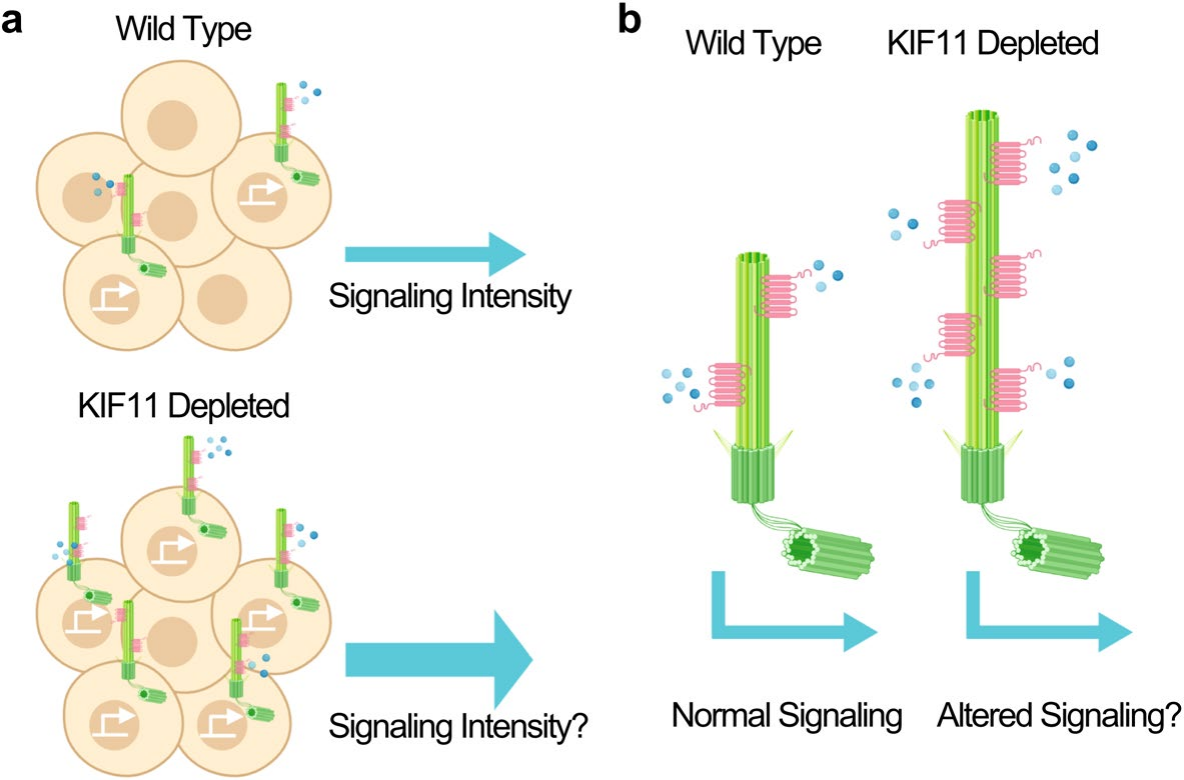
Model of KIF11 regulation of primary cilia dynamics and signaling. (**a**) Model for how increased cilia expression could lead to larger downstream signaling effects. (**b**) Model for how longer cilia could allow for altered receptor density, which in turn could lead to an altered signaling output. Images created with Biorender.com.

Our results are the first to demonstrate that KIF11 is localized at the basal body of primary cilia in multiple cell types, including neoplastic and non-neoplastic cells. Additionally, we have shown a previously unknown ability for KIF11 to regulate primary cilia dynamics. Typically, primary cilia are present when cells are quiescent and are only found on a small percentage of asynchronously growing cells, which are likely in early G1 phase^23^. Our results indicate that by depleting KIF11, we are able to shift this percentage of ciliated cells, implicating KIF11 as a regulator of cilia dynamics. KIF11’s role as a primary cilia-regulator is further supported by our finding that transfection of KIF11 partially rescues the increase in ciliation in asynchronous cells. Further, in heterozygous *KIF11* RPE1 cells, we see the same increase in ciliation. An increase in the amount of ciliated cells is important, as primary cilia regulate the careful balance in signaling patterns required for homeostasis^42–45^. Primary cilia are covered in receptors for different pathways, and oftentimes, these receptors are not expressed anywhere else on the cell^46–48^. Therefore, careful timing of when primary cilia (and therefore, their receptors) are present is critical for proper pathway activity. For example, during neurodevelopment the expansion of the neural progenitor pool relies upon cilia-mediated sonic hedgehog signaling^45^. If there are too many cilia, signaling could be disrupted or aberrant, and conversely, if cells are not ciliated, signaling will not occur. This leads us to consider a model where KIF11 is necessary for proper expression of primary cilia, and may suppress ciliation in inappropriate contexts. Furthermore, if KIF11 is depleted or mutated in a biological system, we hypothesize that there could be an increase in cilia at a time when cells should not assemble cilia. Ultimately, such a loss of suppression of ciliation could lead to aberrant signaling (Fig. 5a). In addition to the number of ciliated cells impacting signaling, the length of the cilium is also important when discussing relative signaling activity. Change in cilium length has been shown to alter signaling as receptor density changes when cilia are shorter and therefore have less surface area to be covered by receptors^39^. Therefore, we hypothesize that the increase in cilium length brought on by changes in KIF11 expression could potentially alter signaling. Similar to shortened cilia having altered signaling abilities, lengthened cilia could impact receptor density or receptor functionality, which may result in abnormal signaling (Fig. 5b)^49,50^. Further, we propose that these impacts on signaling patterns might explain some of the phenotypes seen in patients with MCLMR and FEVR. Future studies could ascertain the role of KIF11 in primary cilia-mediated signaling by examining downstream gene expression in pathways such as sonic hedgehog and Wnt.

Our study is the first to establish a role between KIF11 and regulation of primary cilia, but further exploration will be required to understand the mechanism of how KIF11 impacts primary cilia dynamics. It is worth noting that multiple other mitotic kinesins have been shown to regulate primary cilia, independent of their role in mitosis. KIF14, a kinesin involved in bipolar spindle formation, has recently been shown to also play a role in basal body and primary cilia formation. Upon KIF14 depletion, cilium elongation and assembly is altered, as well as hedgehog signaling, through an association with Aurora Kinase A^51^. KIF2, KIF3, and KIF13 have all been shown to function in mitosis, as well as to have critical primary cilia regulatory roles^52,53^. Additionally, Mps1, a kinase essential for mitosis, has been shown to coordinate with VDAC3 to negatively regulate the inappropriate formation of primary cilia through accumulation at the centrosomes^54^. Perhaps KIF11 is acting in a similar way to one or more of these proteins, and is coordinating with other primary cilia proteins. The cell cycle and primary cilia formation are inextricably linked so it stands to reason, and has been experimentally demonstrated, that many of the proteins involved in the cell cycle might also be critical for the proper formation and regulation of primary cilia. Our data are the first step in exploring KIF11 in this context, and future studies should elucidate the mechanism for this previously unknown role for KIF11.

## Methods

### Cell culture

Human GBM specimen (GBM 08-387; kindly provided by Dr. Jeremy Rich, UC San Diego) was originally isolated from a tumor resection in accordance with approved institutional review board protocols at Duke University and the Cleveland Clinic Lerner Research Institute. GBM 08-387 was maintained through subcutaneous xenografts in the flanks of athymic nude mice or NOD *scid* gamma mice under approved institutional protocols and in accordance with the NIH Guide for the Care and Use of Laboratory Animals. Tumors were dissociated using a Papain Dissociation System (Worthington Biochemical). Cells were cultured in Neurobasal Media (Gibco) with B-27 supplement (without vitamin A; Gibco), basic fibroblast growth factor (10 ng/mL; Gibco), EGF (10 ng/mL; Gibco), l-glutamine (2 mmol/L; Gibco), and sodium pyruvate (1 mmol/L; Gibco) and grown in suspension. Immortalized retinal pigmented epithelial cells (RPE1) were commercially obtained (ATCC) and grown in DMEM/F12 (Gibco) with fetal bovine serum (Gibco). For serum starvation experiments, RPE1 cells were grown in DMEM/F12 without the addition of fetal bovine serum. Immortalized human neural progenitors were obtained commercially (SigmaMillipore) and were grown in ReNcell neural stem cell maintenance media (SigmaMillipore) with basic fibroblast growth factor (10 ng/mL; Gibco) and EGF (10 ng/mL; Gibco). All cells were cultured at 37 °C at 5% CO_2_. For cell counting before each experiment, a single-cell suspension was achieved using TrypLE (Gibco). Mycoplasma testing was done quarterly (LookOut Mycoplasma PCR Detection Kit; Sigma-Aldrich) and cell line verification was done annually (microsatellite genotyping; OSUCCC Genomics Shared Resource).

### Immunofluorescence

Tumorspheres were fixed in 100% methanol and immunolabeled with combinations of the following primary antibodies: anti-Arl13b (Proteintech; 17711-1-AP; rabbit; 1:250), anti-Cep135 (Abcam; ab75005; rabbit; 1:1,000), anti-KIF11 (BD Biosciences; 611,186; mouse; 1:500), anti-pericentrin (Covance; PRB-432C; rabbit; 1:500), anti-Ki67 (Cell Signaling; 9,449; mouse; 1:800), or anti-Sox2 (R&D Systems; MAB2018; mouse; 1:100) overnight at 4 °C. Primary labeling was followed by secondary detection with Alexa Fluor 488 and 568 (Invitrogen; 1:1,000) for 1 h at room temperature. Nuclei were counterstained with Hoechst. Tumorspheres were resuspended in Fluoromount-G (SouthernBiotech) and mounted onto slides. Images were taken using a Zeiss LSM 800 confocal microscope. RPE1 cells and neural progenitor cells were plated onto glass coverslips and fixed in 100% methanol, permeabilized in PBS-Triton X-100 (0.2% vol/vol) or rehydrated with PBS, and immunolabeled with anti-Arl13b, anti-Cep135, anti-acetylated tubulin (Sigma; T 6,793; mouse; 1:2,000), anti-KIF11 for 1 h at room temperature, followed by secondary detection with Alexa Fluor 488, 568, and 647 (Invitrogen; A32731, A-11031, A32733; goat α-rabbit, goat α-mouse, goat α-rabbit; 1:500 or 1:1,000) for 1 h at room temperature. Nuclei were counterstained with Hoechst. Coverslips were then mounted onto slides using Fluoromount-G (SouthernBiotech). Images were taken using a Zeiss LSM 800 confocal microscope or a Leica DM5500B upright epifluorescence 171 microscope.

### KIF11 depletion

For siRNA experiments, RPE1 cells were plated at 100,000 cells per well of a 6 well plate containing glass coverslips. 24 h after plating, cells were transfected with either siControl, siKIF11-1 (both to a final concentration of 5 nM), or siKIF11-2 (to a final concentration of 250 pM) using RNAimax transfection reagent (Thermo Fisher) according to the RNAimax protocol. Media was changed every 24 h, and cells were continuously monitored to ensure no gross mitotic arrest or cell death. At 72 h post siRNA transfection, cells were fixed with methanol for immunofluorescence or harvested for immunoblotting to ensure KIF11 depletion. siRNAs were obtained from Integrated DNA Technologies IDT; (siKIF11-1: AGTTTAGAGACATCTGACTTTGATAGCTAAATTAAA, siKIF11-2: GGCAAAAACCTGAATAGTCTGTTTA). For rescue experiments, 24 h after siRNA addition, 0.5–1 µg of pEGFP or pEGFP-KIF11 DNA plus 1–1.5 µg pcDNA3.1 carrier DNA were transfected to the cells using Fugene 6 transfection reagent and associated protocol. Media was changed 24 h later, and cells were collected 24 h after media change. CRISPR clones were generated by using the ribonucleoprotein (RNP) method. This method refers to the use of a guide RNA which is complexed to purified Cas9 protein. All products used to generate CRISPR clones were obtained from IDT. crRNA and tracrRNA were complexed together to create the gRNA; the crRNA is KIF11-specific and was designed by IDT (CGTGGAATTATACCAGCCAA). The gRNA and purified Cas9 were combined to create the RNP complexes, and were delivered to RPE1 cells via transfection with CRISPRmax and Cas9 Plus transfection reagents. After transfection with RNPs, cells were incubated for 48 h. After 48 h, cells were plated sparsely (20 cells per well of a 6 well plate). Cells were monitored over the next two weeks to watch for clonal outgrowth. Individual colonies were then harvested and moved into their own well of a 96 well plate. These colonies were expanded for approximately 2 more weeks, at which point cells were harvested for immunoblotting to detect levels of KIF11 and expanded for experimental use.

### GFP and GFP-KIF11 overexpression

RPE1 cells were plated at 100,000 cells per well of a six well plate containing glass coverslips. Twenty-four hours later, 0.5 µg of pEGFP or pEGFP-KIF11 DNA plus 1.5 µg pcDNA3.1 carrier DNA were transfected to the cells using Fugene 6 transfection reagent and associated protocol. Cells were either collected and fixed at 24 h or had media changed at 24 h and were collected at 48 h.

### Cilium quantification and measurement

Percentage of ciliated cells was quantified by imaging experimental cells, and counting the total number of cells and the number of ciliated cells. Percentages were calculated by number of ciliated cells/total number of cells. When GFP transfection was used, only GFP-positive cells were used in the calculation of ciliated cells/total cells. Cilia length was measured for siKIF11 experiments with the Zen (blue edition) software compatible with Zeiss microscopes. Cilia were traced with the measurement tool to calculate their length in microns. The same measurement was conducted in the crKIF11 cells, but using the LAS AF software, compatible with Leica microscopes.

### Protein extraction and immunoblotting

Whole cell extracts were made using a 50 mM Tris pH 8.0, 120 nM NaCl, 0.5% NP-40 lysis solution supplemented with protease and phosphatase inhibitors (Roche). Samples were run on 10% SDS-PAGE gels and transferred to PVDF membranes (Millipore Corp.). The membranes were blocked with 5% (w/v) dry milk in TBS-Tween-20 (TBST; 0.1–0.2% v/v) and probed with primary antibodies overnight at 4 °C (anti-KIF11, BD Biosciences, 611,186, mouse, 1:3,000; anti-ßactin, Sigma, A1978, mouse, 1:20,000). Secondary antibodies (LI-COR Biosciences, 926-68070, goat α-mouse, 1:20,000) were incubated in TBST plus 0.01–0.02% SDS and visualized with the LI-COR Odyssey near infrared imaging system.

### Immunoprecipitation

Immunoprecipitations were carried out using Dynabeads Protein G (Invitrogen). Briefly, 25 µL of beads were incubated with 3 µg of KIF11 antibody (Novus; NB500-181; rabbit) for 30 min at room temperature followed by washes and then an overnight incubation at 4 °C with 25 mg of G0 synchronized RPE1 whole cell extract. For analysis of proteins off beads, immunoprecipitated proteins were released from the beads by boiling in Laemmli 2X concentrate sample buffer (Sigma).

### Samples for LC–MS/MS analysis

For “on-bead” analysis, KIF11 from G0 synchronized RPE1 was immunoprecipitated as described above with resulting immmunoprecipitated protein taken on beads for processing by the Proteomics Shared Resource at The Ohio State University Comprehensive Cancer Center for protein quantification and identification of protein interactors. For KIF11 that was boiled off beads, samples were resolved on 4–20% Mini-PROTEAN TGX precast gels (Bio-Rad Laboratories, Inc.) and visualized with GelCode Blue Stain Reagent as per manufacturer instructions (ThermoFisher Scientific). The KIF11 band was then cut from the gel and processed by the Lerner Research Institute Mass Spectrometry Laboratory for Protein Sequencing for proteomic analysis and identification of interacting proteins.

### Cell growth

Cell growth was monitored after depletion of KIF11 by EdU labeling and by Incucyte ZOOM live-cell imaging. EdU labeling was accomplished by exposing cells to a 4-h pulse of EdU prior to harvesting the cells at the end of the experiment. Cells that were exposed to EdU were then fixed in 100% methanol, permeabilized in PBS-Triton X-100 (0.2% vol/vol), and labeled with the reagents and protocol associated with the Click-iT EdU Cell Proliferation Kit for Imaging (Thermo Fisher). Nuclei were counterstained with Hoechst. Coverslips were then mounted onto slides using Fluoromount-G (SouthernBiotech). Images were taken using a Zeiss LSM 800 confocal microscope. To track cells via Incucyte live-cell imaging, 50,000 WT RPE1 or crKIF11 cells were plated in wells of a 6 well plate and placed on the Incucyte. Cell confluence was used as a measure of growth, and was monitored over 40 h. Multiple areas of each well were monitored.

### Statistical analyses

Statistical significance was calculated with GraphPad Prism Software using a Student’s *t*-test or 2-way ANOVA with Sidak’s post-test (GraphPad Software, Inc.).

## Supplementary information

Supplementary Information.
