# Supplementary material for: An interphase pool of KIF11 localizes at the basal bodies of primary cilia and a reduction in KIF11 expression alters cilia dynamics
Source: Sci Rep. 2020 Aug 18;10:13946. doi: 10.1038/s41598-020-70787-4 (PMC7434902; doi:10.1038/s41598-020-70787-4)
Supplement: Supplementary file 1 — Supplementary Information. [file 41598_2020_70787_MOESM1_ESM.pdf]

An interphase pool of KIF11 localizes at the basal bodies of primary cilia and a reduction in KIF11 expression alters cilia dynamics

Abigail A Zalenski, Shubhra Majumder, Kuntal De, Monica Venere

Supplementary Figure 1. KIF11 is depleted by siRNA without compromising cell cycle progression and overexpressing KIF11 does not impact ciliogenesis.

a) siControl, siKIF11-1, and siKIF11-2 lysates from asynchronously growing cells were probed with α-KIF11. βactin served as a loading control. The molecular weight (MW) of resulting bands is given in kilodaltons (kDa). Lines indicate that non-relevant lanes were removed from the image. b) Full image of siRNA immunoblot used in a. Lane 1 is wild type lysate, lane 3 is siKIF11-2, lane 6 is siKIF11-1 and are denoted with asterisks. c) Cells treated with siControl or siKIF11-1 were exposed to EdU and then fixed. Cells were labeled for EdU and DNA was counterstained with Hoechst. Percent of EdU positive cells was calculated for each condition and graphed (n=3 biological replicates performed, 90-150 cells per replicate). Error bars represent standard deviation. d) Cells were transfected with either GFP or GFP-tagged KIF11. At 24 hours and 48 hours post transfection cells were collected and fixed. Cells were labeled for α-GFP (green), α-Arl13B (red), α-Cep135 (red), and DNA was counter-stained with Hoechst. Percent of GFP-positive cells with cilia were calculated for each group and graphed (n=3 biological replicates performed, 50-100 cells per replicate). Error bars represent standard deviation.

Supplementary Figure 2. KIF11 is depleted by CRISPR/Cas9 gene editing without compromising cell cycle progression.

a) Wild type (WT) RPE1 and crKIF11 lysates from asynchronously growing cells were probed with α-KIF11. βactin served as a loading control. The molecular weight (MW) of resulting bands is given in kilodaltons (kDa). Lines indicate that non-relevant lanes were removed from the image. b-c) Full images of CRISPR/Cas9 immunoblots used in a. KIF11 (b) and βactin (c) were probed sequentially. Lane 1 is wild type lysate, lane 3 is crKIF11-1, and lane 5 is crKIF11-2 and are denoted with asterisks. d) WT RPE1, crKIF11-1, and crKIF11-2 cells were all exposed to EdU and then fixed. Cells were labeled for EdU and DNA was counterstained with Hoechst. Percent of EdU positive cells was calculated for each condition and graphed (n=3 biological replicates performed, 100-150 cells per replicate). Error bars represent standard deviation. e) Growth of WT RPE1 and crKIF11 cells were monitored on the Incucyte ZOOM live-cell imaging system over 40 hours. Percent confluence was used to monitor growth rate with multiple areas per well monitored and averaged.

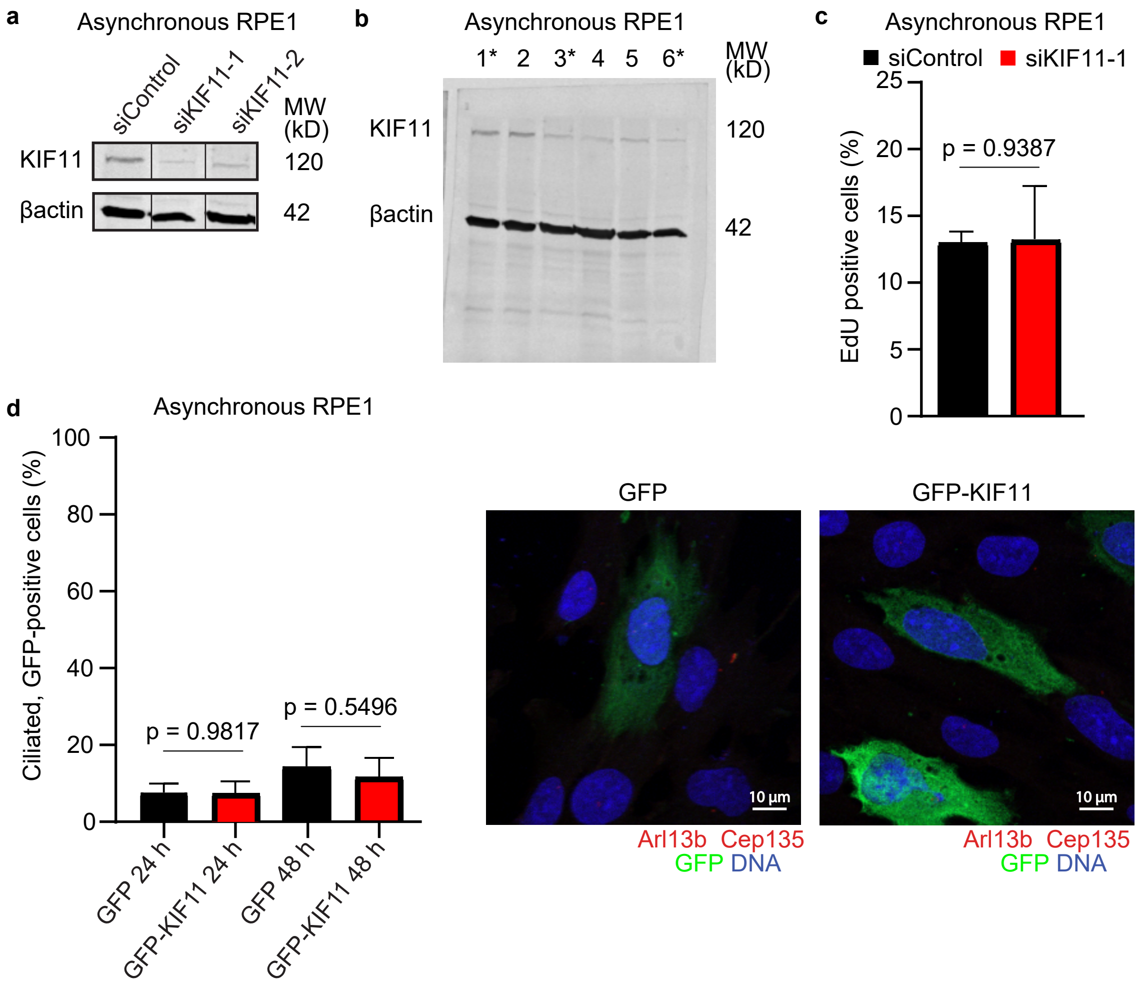

a
Asynchronous RPE1
siControl
siKIF11-1
siKIF11-2
MW (kD)
KIF11
120
βactin
42
b
Asynchronous RPE1
1* 2 3* 4 5 6*
MW (kD)
KIF11
120
βactin
42
c
Asynchronous RPE1
siControl
siKIF11-1
EdU positive cells (%)
25
20
15
10
5
0
p = 0.9387
d
Asynchronous RPE1
Ciliated, GFP-positive cells (%)
100
80
60
40
20
0
p = 0.9817
p = 0.5496
GFP 24 h
GFP-KIF11 24 h
GFP 48 h
GFP-KIF11 48 h
GFP
GFP-KIF11
10 µm
10 µm
Arl13b Cep135
GFP DNA
Arl13b Cep135
GFP DNA

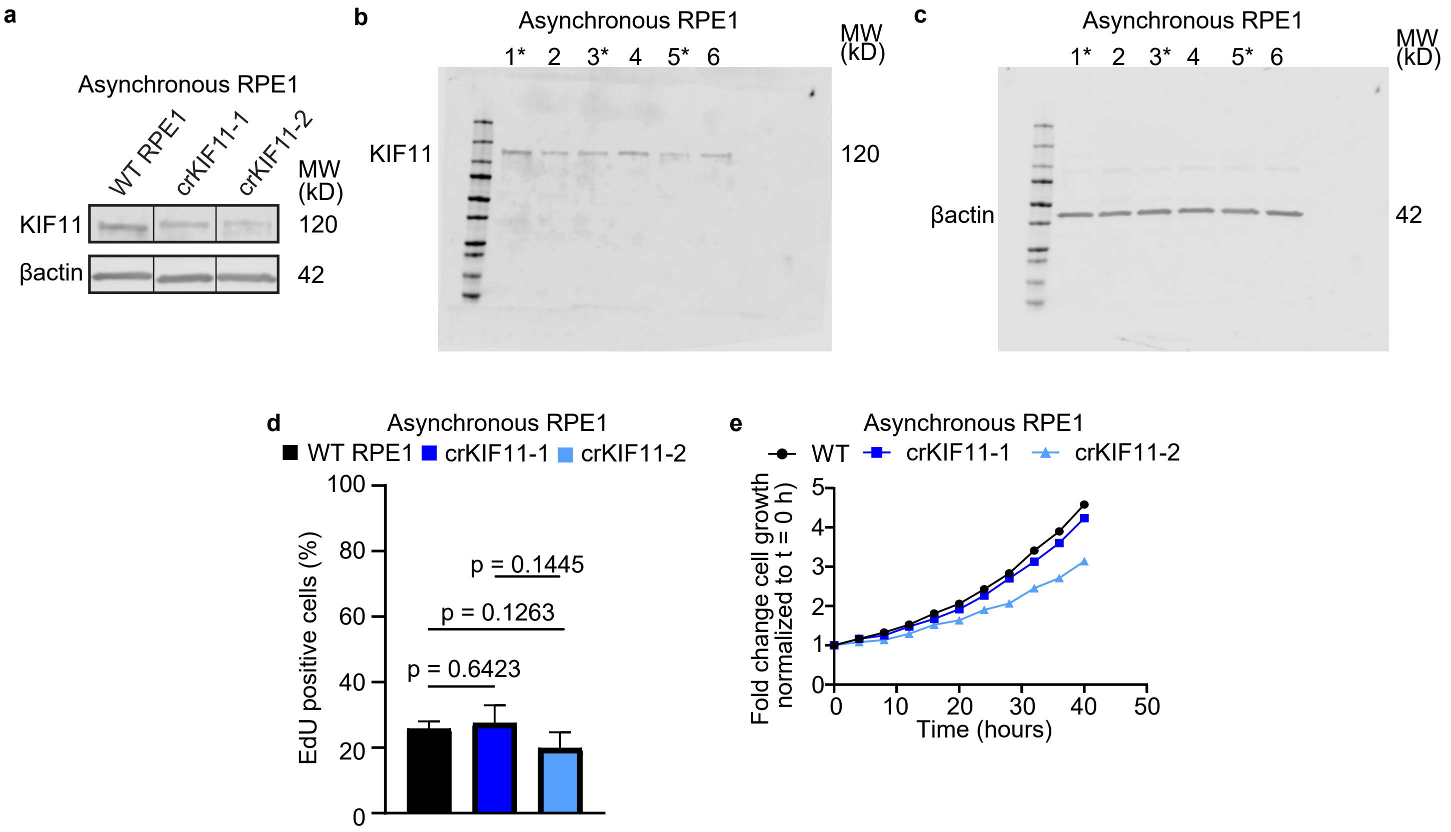

a
Asynchronous RPE1
WT RPE1
crKIF11-1
crKIF11-2
MW (kD)
KIF11
120
βactin
42
b
Asynchronous RPE1
1* 2 3* 4 5* 6
MW (kD)
KIF11
120
c
Asynchronous RPE1
1* 2 3* 4 5* 6
MW (kD)
βactin
42
d
Asynchronous RPE1
WT RPE1
crKIF11-1
crKIF11-2
EdU positive cells (%)
p = 0.1445
p = 0.1263
p = 0.6423
0
20
40
60
80
100
e
Asynchronous RPE1
WT
crKIF11-1
crKIF11-2
Fold change cell growth normalized to t = 0 h)
Time (hours)
0
10
20
30
40
50
0
1
2
3
4
5
